# Interspecies-Extrapolated Biotic Ligand Model to Predict Arsenate Toxicity to Terrestrial Plants with Consideration of Cell Membrane Surface Electrical Potential

**DOI:** 10.3390/toxics10020078

**Published:** 2022-02-08

**Authors:** Jinsung An

**Affiliations:** Department of Biological and Environmental Engineering, Semyung University, 65 Semyung-ro, Jecheon-si 27136, Korea; jsan@semyung.ac.kr; Tel.: +82-43-649-1335; Fax: +82-43-649-1779

**Keywords:** acute toxicity prediction model, species specificity, inherent sensitivity, environmental modulator, root cell plasma membrane surface electrical potential

## Abstract

Arsenic is a metalloid that is highly toxic to living organisms in the environment. In this study, toxicity caused by inorganic arsenate (As(V)) to terrestrial plants, such as barley *Hordeum vulgare* and wheat *Triticum aestivum*, was predicted using the existing biotic ligand model (BLM) for bioluminescent *Aliivibrio fischeri* via interspecies extrapolation. Concurrently, the concept of cell plasma membrane electrical potential (Ψ_0_) was incorporated into the extrapolated BLM to improve the model predictability in the presence of major cations such as Ca^2+^. The 50% effective As(V) toxicity (EC_50_{HAsO_4_^2−^}) to *H. vulgare* decreased from 45.1 ± 4.34 to 15.0 ± 2.60 µM as Ca^2+^ concentration increased from 0.2 to 20 mM owing to the accumulation of H_2_AsO_4_^−^ and HAsO_4_^2−^ on the cell membrane surface. The extrapolated BLM, which only considered inherent sensitivity, explained well the alteration of As(V) toxicity to *H. vulgare* and *T. aestivum* by Ca^2+^ with in an order of magnitude, when considering a linear relationship between Ψ_0_ and EC_50_{HAsO_4_^2−^}.

## 1. Introduction

Arsenic (As) accumulates in environmental media via both natural and anthropogenic sources and can cause severe adverse effects on human health as well as ecosystems [1,2,3,4,5]. Because the total As concentration in the environmental media cannot accurately indicate bioavailability or ecotoxicity, it is important to rationally assess the ecotoxicity of As using suitable bioassays [6]. The ecotoxicity of As varies greatly depending on environmental factors (e.g., phosphate concentration) based on which the bioassay is performed [7,8] and on the sensitivity of the species tested [9]. The environmental factors can be considered using the biotic ligand model (BLM), which is a semi-mathematical and equilibrium model that predicts site-specific toxicity of cationic metals such as Cd, Cu, Ni, Pb, and Zn [10,11,12,13]. An et al. [14] recently developed a BLM for inorganic arsenate (As(V)), which is a predominant species in surface water and surface soil porewater. This BLM can effectively predict As(V) toxicity to the bioluminescence of *Aliivibrio fischeri* considering phosphate competition (toxicity alleviation) and As species alteration by pH. The sensitivity of the species tested can be considered using species sensitivity distribution (SSD), which is the cumulative distribution of toxicity of a single compound to a set of species that constitutes a community [15]. SSD can estimate the 5% hazardous concentration (HC5), which can be defined as the total dissolved concentration of the contaminant that protects 95% of the species in the ecosystem.

To simultaneously overcome site-specificity and species-specificity while assessing ecotoxicity, thereby deriving a safe concentration (e.g., HC5) in the ecosystem by combining BLM and SSD, the full SSD bioavailability normalization approach [16,17,18,19] can be used. In this approach, HC5 is estimated using an SSD obtained from the entire normalized (i.e., site-specific physicochemical properties that can affect the toxicity of metals or metalloids determined using the BLM) ecotoxicity data set. This approach also assumes that some species likely share the BLM parameters, that is, conditional binding constants between free metal ions or major cations and active binding sites (ABSs; i.e., biotic ligand) at the cell membrane surface of the organism [19]. This assumption has been supported by the fact that BLMs for Cu, Ni, and Zn can be extrapolated across species [16,17,18,20].

To the best of our knowledge, the BLM for As(V) only considers the effect of phosphate competition on *A. fischeri*, as suggested in a previous study [14], and not on individual species. Moreover, evidence supporting extrapolation with respect to the BLM for As(V) is still scarce. Therefore, in this study, the feasibility of interspecies extrapolation was tested to extend the BLM for As(V) to terrestrial plants.

The phenomenon through which major cations alleviate metal toxicity can be explained in terms of BLM, as well as the concept of cell plasma membrane (PM) electrical potential (Ψ_0_) [21,22,23]. The root cell PM surface, which is negatively charged, attracts cations in the bulk-phase medium. The addition of major cations, such as Ca^2+^, to the solution increases Ψ_0_ and decreases the cationic free metal ions moving to the cell PM surface via electrostatic attraction [21]. In contrast, As(V), which is normally present as H_2_AsO_4_^−^ and HAsO_4_^2−^, can accumulate on the PM surface. Wang et al. [22] revealed a significant increase in As(V) toxicity to wheat (*Triticum aestivum*) with increasing Ca^2+^ activity in the bulk solution. This was because the increase in Ca^2+^ activity from 0.2 to 2.5 mM in the solution increased Ψ_0_ from −40 to −20 mV, resulting in an increased accumulation of H_2_AsO_4_^−^ and HAsO_4_^2−^ on the root cell PM surface of *T. aestivum*. However, such an increase in As(V) toxicity cannot be explained using the current BLM for As(V) [14].

The aim of this study was to confirm that the BLM for As(V), which was originally developed using *A. fischeri*, can predict As(V) toxicity to terrestrial plant species (i.e., *Hordeum vulgare* and *T. aestivum*) by optimizing the inherent sensitivity (IS). In this regard, the feasibility of interspecies extrapolation of BLM for As(V) was evaluated, and the predicted and measured 50% effective As(V) activities (EC_50_{HAsO_4_^2−^}) were compared to assess the predictability of BLM for As(V) after adjusting for the inherent sensitivity (IS) of plants. Furthermore, a methodology was established to incorporate the concept of Ψ_0_ into the extrapolated BLM for As(V) for considering the increased toxicity of As(V) by Ca^2+^.

## 2. Materials and Methods

### 2.1. Toxicity Test

The root growth inhibition test using barley *H. vulgare* was performed according to ISO guideline 11269-1 [24] with some modifications. Seeds were sterilized in a 5% NaOCl solution (Daejung, Korea) for 10 min and rinsed three times with deionized (DI) water, with a resistance of 18.2 MΩ cm (Milli-Q, Millipore, Bedford, MA, USA). The seeds were then germinated on filter paper moistened with DI water for 36 h at room temperature (25 °C) in complete darkness. Each germinated seed was fixed on a polyethylene sheet that floated on the medium surface. For each medium, a toxicity test was conducted, comprising seven treatments (control + six different concentrations of As(V)). Each treatment was performed using six germinated seeds in a polyethylene beaker filled with 500 mL of the test medium as a hydroponic culture. All test media were incubated for 5 days at 20 °C with a light:dark cycle of 16:8 in a growth chamber (E15, Conviron, Winnipeg, Canada). The toxicity of As(V), measured as the relative root elongation (RRE), after exposure to a given concentration of As(V) was calculated [25] as follows:

(1) $$\mathrm{RRE}=100\times \frac{L_{f,t}-L_{i,t}}{L_{f,c}-L_{i,c}}$$

where RRE is the relative root elongation (%), L_f,t_ is the average root length (mm) of each treatment after 5 days of exposure, L_i,t_ is the average root length (mm) of each treatment at the initial time (t = 0), L_f,c_ is the average root length (mm) of the control after 5 days of exposure, and L_i,c_ is the average root length (mm) of the control at the initial time.

The changes in the RRE in response to the changes in As(V) concentrations or activities were fitted to a sigmoidal dose–response curve (Equation (2)) to calculate the 50% effective concentration (EC50).

(2) $$y=y_{0}+\frac{a}{1+e^{-\frac{x-x_{0}}{b}}}$$

where y is the observed RRE; x is the natural logarithm of the exposed As(V) concentration or activity; a, b, x_0_, and y_0_ are the fitting parameters that determine the shape of the dose–response curve.

### 2.2. Effect of Ca^2+^ Concentration on Arsenate Toxicity to H. vulgare

All reagents used in this study were of analytical grade. DI water was used throughout. A stock solution of As(V) (10 mM) was prepared by dissolving Na_2_HAsO_4_·7H_2_O (98.0–102%, Sigma–Aldrich) in DI water and stored in the dark at 4 °C prior to use. The As(V) concentrations in each test medium (i.e., in direct contact with *H. vulgare*) were 0, 0.5, 2, 10, 50, 200, and 1000 μM. The pH of each test medium was adjusted to 7 ± 0.05 using 3.6 mM 3-[N-morpholino] propanesulfonic acid (MOPS, >99.5%, Sigma–Aldrich), by adding 1 M NaOH or 1 M HCl. Background concentrations of major cations and anions in each test medium were as follows: Ca^2+^ = 0.2 mM, Mg^2+^ = 0.05 mM, K^+^ = 0.2 mM, Na^+^ = 1 mM, PO_4_^3−^ = 0.05 mM, SO_4_^2−^ = 0.05 mM, and NO_3_^−^ = 0.2 mM. The independent effect of Ca^2+^ on As(V) toxicity to *H. vulgare* was assessed at pH 7 in 3.6 mM MOPS buffer. Each set comprised a series of solutions with five different Ca^2+^ concentrations (i.e., 0.2, 1, 5, 10, and 20 mM).

### 2.3. Chemical Analysis

The concentrations of major cations and total As were determined via inductively coupled plasma atomic emission spectrometry (ICP-AES) (ICAP 7400 DUO, Thermo Scientific, Waltham, MA, USA). The concentrations of the major anions were determined via ion chromatography (DX500, Dionex, Sunnyvale, CA, USA). Preliminary tests revealed that the measured concentrations did not differ significantly from their nominal values within 5% variability. Since the salt of pentavalent arsenate (i.e., Na_2_HAsO_4_·7H_2_O) was dissolved in DI water to prepare As(V) stock solution in this study, the total As concentration determined using ICP-AES was considered as the concentration of As(V). In order to validate this assumption, the test medium containing 50 μM As was stored 5 days, and then analyzed using high performance liquid chromatography (1260 In-finity LC system, Agilent Technologies, Santa Clara, CA, USA) linked to inductively coupled plasma mass spectrometry (7700x, Agilent Technologies, Santa Clara, CA, USA). It was confirmed that As(V) species did not alter significantly within 5% varia-bility.

Visual MINTEQ 3.1 [26] was used to calculate As(V) activities (H_2_AsO_4_^−^ and HAsO_4_^2−^) and the major cations/anions in the tested solution. Temperature, pH, ion concentrations, and partial pressure of CO_2_ (P_CO2_ = 0.00038 atm) were the input data.

The analysis of variance (ANOVA) was performed using Microsoft Excel 2010 Analysis ToolPak to evaluate whether Ca^2+^ significantly (*p* < 0.05) affected As(V) toxicity.

### 2.4. Calculation of the Electrical Potential of Root Cell PM Surface

To calculate Ψ_0_ and the corresponding ion activities at the PM surface, the Gouy–Chapman–Stern (GCS) model [27] was used. The GCS model comprises two parts: (i) electrostatic theory (Gouy–Chapman theory) and (ii) interaction between ions and the PM surface (Stern portion). The calculation process is described as follows:

First, the actual PM surface charge density (σ, unit: C/m^2^) can be described using Equation (3):

(3) $$\sigma^{2}=2\varepsilon_{r}\varepsilon_{0}\mathrm{RT}\sum_{i}{\left[I^{z}\right]}_{b}(\exp[\frac{-Z_{i}{F\Psi }_{0}}{\mathrm{RT}}]-1)\;$$

where $2\varepsilon_{r}\varepsilon_{0}$ RT = 0.00345, when the concentrations are expressed in units of mol/L at 25 °C; ε_r_ is the dielectric constant of water; ε_0_ is the permittivity of vacuum; F, R, and T are the Faraday constant, gas constant, and absolute temperature, respectively; [I^Z^]_b_ is the free ion concentration in the bulk solution; Z_i_ is the charge of the ith ion.

Second, if the PM surface binding sites comprising negatively charged sites (R^−^) and neutral sites (P^0^) are occupied by ions (I^Z^), the PM surface species and their equilibrium reactions can be represented as Equations (4) and (5):

(4) $$K_{\mathrm{RI}}=\frac{\left[{\mathrm{RI}}^{Z-1}\right]}{\left[R^{-}\right]{\left[I^{Z}\right]}_{0}}$$

(5) $$K_{\mathrm{PI}}=\frac{\left[{\mathrm{PI}}^{Z}\right]}{\left[R^{0}\right]{\left[I^{Z}\right]}_{0}}$$

where [R^−^], [P^0^], [RI^Z−1^], and [PI^Z^] denote the PM surface densities expressed in mol/m^2^; and [I^Z^]_0_ denotes the concentration of the unbound I^Z^ ion at the PM surface.

Finally, σ in the Graham equation (Equation (3)) can be expressed by Equation (6) as follows:

(6) $$\sigma =\left\{-\left[R^{-}\right]+\sum_{i}\left(Z_{i}-1\right)\left[{\mathrm{RI}}^{Z-1}\right]+\sum_{i}\left(Z_{i}\right)\left[{\mathrm{PI}}^{Z}\right]\right\}F$$

To calculate the values of [I^Z^]_0_, the Boltzmann equation (Equation (7)) can be used:

(7) $${\left[I^{z}\right]}_{0}={\left[I^{z}\right]}_{b}\exp\left(\frac{-Z_{i}{F\Psi }_{0}}{\mathrm{RT}}\right)$$

To determine Ψ_0_, the trial values were assigned to Ψ_0_ in Equations (3)–(7) until the values of σ computed in Equations (3)–(6) converge. The equilibrium constants and total surface densities of the binding sites R and P in Equations (4) and (5) were previously determined and are available from Kopittke et al. [27].

### 2.5. Interspecies Extrapolation of BLM

The BLM for predicting As(V) toxicity to *A. fischeri* [14] is presented in Equation (8):

(8) $${\mathrm{EC}}_{50}\left\{{\mathrm{HAsO}}_{4}{}^{2-}\right\}=\frac{f_{\mathrm{mix}}^{50\%}\left[1+K_{\mathrm{XH}2\mathrm{PO}4}\left\{H_{2}{\mathrm{PO}}_{4}{}^{-}\right\}+K_{\mathrm{XHPO}4}\left\{{\mathrm{HPO}}_{4}{}^{2-}\right\}\right]}{\left(1-f_{\mathrm{mix}}^{50\%}\right)\left[\frac{K_{\mathrm{XH}2\mathrm{AsO}4}}{K_{\mathrm{As}}}\left\{H^{+}\right\}+K_{\mathrm{XHAsO}4}\right]}$$

where K_XA_ is the conditional binding constant for As(V) or phosphate bound to the ABS (L/mol); {A^n−^} is the activity of As(V) or phosphate (mol/L) in the bulk solution; K_As_ is the acid dissociation constant of As(V) (pK_As_ = 6.76); f_mix_^50%^ is the ABS required to be occupied by As(V) (both H_2_AsO_4_^−^ and HAsO_4_^2−^) for inducing 50% toxicity [14]. The BLM parameters, including the conditional binding constants and f_mix_^50%^, are presented in Table 1a.

(9) $$\mathrm{EC}50_{\mathrm{site}}=\mathrm{IS}\times {\mathrm{EM}}_{\mathrm{site}}$$

(10) $$\mathrm{IS}=\frac{\mathrm{EC}50_{\mathrm{test}}}{{\mathrm{EM}}_{\mathrm{test}}}=\frac{f_{\mathrm{mix}}^{50\%}}{1-f_{\mathrm{mix}}^{50\%}}$$

(11) $$\mathrm{EM}=\frac{1+K_{\mathrm{XH}2\mathrm{PO}4}\left\{H_{2}{\mathrm{PO}}_{4}{}^{-}\right\}+K_{\mathrm{XHPO}4}\left\{{\mathrm{HPO}}_{4}{}^{2-}\right\}}{\frac{K_{\mathrm{XH}2\mathrm{AsO}4}}{K_{\mathrm{As}}}\left\{H^{+}\right\}+K_{\mathrm{XHAsO}4}}$$

The site-specific EC50 of *H. vulgare* was calculated by multiplying the IS of the tested species and the environmental modulator (EM) [19]. IS was derived from the measured EC50 and chemical analysis data of the media (i.e., phosphate activities and pH) in toxicity tests. Hence, to determine the IS (corresponding f^50%^_mix_ value), a single EC50 of *H. vulgare* for As(V) obtained from the toxicity test was required.

## 3. Results and Discussion

### 3.1. Effect of Increasing Ca^2+^ Concentrations on As(V) Toxicity

**Figure 1:**
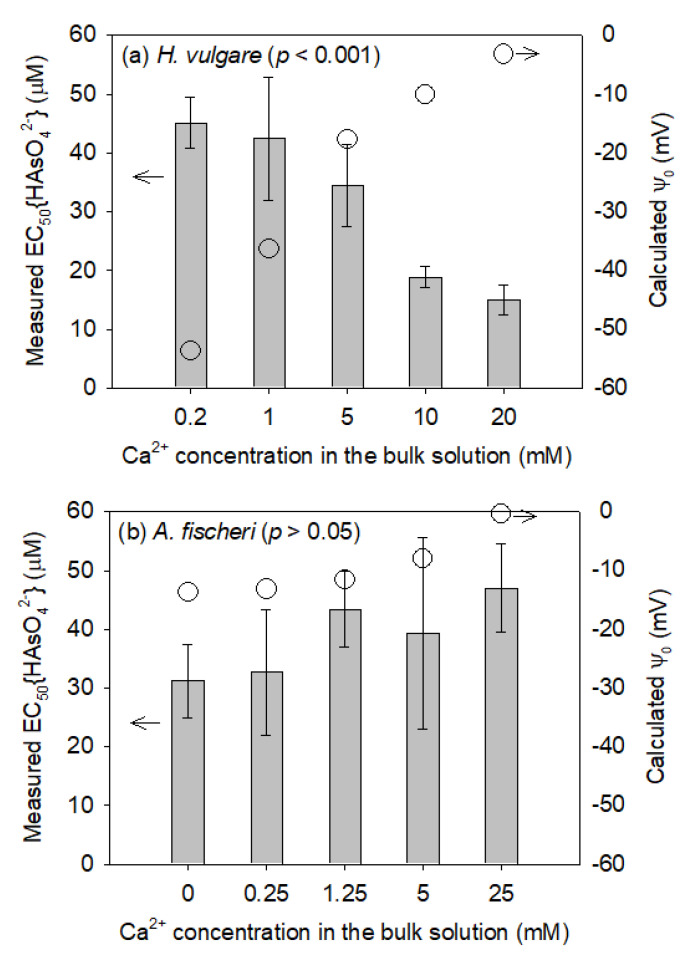
Measured EC50 values expressed as HAsO_4_^2−^ activity (left *y*-axis) obtained from (**a**) root elongation tests using *Hordeum vulgare* and (**b**) bioluminescence inhibition tests using *Aliivibrio fischeri* (data obtained from An et al. [14]). Calculated cell membrane surface electrical potential (Ψ_0_) (right *y*-axis) for (**a**) *H. vulgare* and (**b**) *A. fischeri* with varying Ca^2+^ concentrations. Solid bars and error bars indicate the EC50 values and their standard deviations (*n* = 3), respectively. Open circles represent the calculated Ψ_0_ values. The *p*-value of the ANOVA test was >0.05, indicating that Ca^2+^ addition did not significantly affect As(V) toxicity.

The changes in the EC_50_{HAsO_4_^2−^} values with varying Ca^2+^ concentrations are shown in Figure 1. The EC_50_{HAsO_4_^2−^} of *H. vulgare* significantly decreased from 45.1 ± 4.34 to 15.0 ± 2.60 μM as the Ca^2+^ concentrations increased from 0.2 to 20 mM (Figure 1a). However, the EC_50_{HAsO_4_^2−^} of *A. fischeri*, which was the species used for developing the original BLM for As(V), was not significantly altered, although the Ca^2+^ concentrations increased from 0 to 25 mM (Figure 1b) [14]. This may be due to the sharp increase in Ψ_0_ of *H. vulgare* from −53.8 to −3.3 mV. However, only a slight increase in Ψ_0_ from −13.7 to −0.4 mV was obtained for *A. fischeri* because the experimental solution containing 0.342 M NaCl was completely used for the osmotic pressure control in the toxicity test using *A. fischeri* and, thus, the solution already had a considerable number of positive ions. As reported in previous studies [21,22,23], an increase in Ψ_0_ accumulates more H_2_AsO_4_^−^ and HAsO_4_^2−^, thereby increasing As(V) toxicity. The EC_50_{HAsO_4_^2−^} of *H. vulgare* decreased 3.02 times, indicating its significance for more accurate prediction and evaluation of As(V) toxicity; however, it cannot be obtained using the current BLM for As(V).

**Figure 2:**
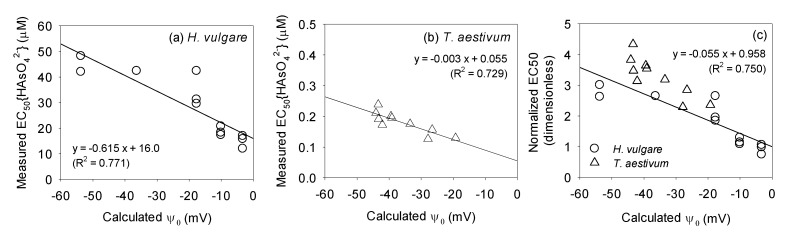
Measured EC50 values expressed as HAsO_4_^2−^ activity from root elongation tests using (**a**) *H. vulgare* conducted in this study and (**b**) *T. aestivum* obtained from Wang et al. [22] plotted against calculated PM surface electrical potential (Ψ_0_). Normalized EC50 calculated by dividing the measured EC_50_{HAsO_4_^2−^} by the *y*-axis intercept (i.e., a situation where Ψ_0_ = 0) of the negative linear relationship of each species presented in Figure 2a,b is also plotted against Ψ_0_ (**c**) to quantify the effect of Ψ_0_ by eliminating species sensitivity. The solid line represents the linear regression curve.

A negative linear relationship (slope = −0.615, R^2^ = 0.771) between the calculated Ψ_0_ and measured EC_50_{HAsO_4_^2−^} of *H. vulgare* is shown in Figure 2a. Wang et al. [22] revealed a similar trend in wheat *T. aestivum* (slope = −0.003, R^2^ = 0.729; Figure 2b). The slopes of the linear relationships between the calculated Ψ_0_ and measured EC_50_{HAsO_4_^2−^} were different owing to the differences in species sensitivity to As(V). Therefore, the normalized EC50, which can be defined as the measured EC_50_{HAsO_4_^2−^} divided by the *y*-axis intercept of the negative linear relationship (i.e., a situation where Ψ_0_ = 0), was used to quantify the effect of Ψ_0_ when compensating for species sensitivity (Figure 2c). Herein, the same negative linear relationship (slope = −0.055, R^2^ = 0.742) was obtained for both species (*H. vulgare* and *T. aestivum*). This indicates that the influence of Ψ_0_ is not a plant-specific property but a physicochemical property based on the attraction or repulsion of As(V) with the PM surface. Consequently, the effect of Ψ_0_ could be considered in a similar way for various plants that have a negatively charged PM surface.

### 3.2. Interspecies Extrapolation of BLM to Predict As(V) Toxicity to H. vulgare and T. aestivum

To extrapolate the BLM originally developed from *A. fischeri* to barley *H. vulgare*, the IS (f_mix_^50%^) had to be determined first. The EC50 value (i.e., EC_50_{HAsO_4_^2−^}) of *H. vulgare* was 45.1 ± 4.34 μM, when the concentrations of major cations and anions were set to background levels by adjusting the pH to 7. Using the calculated EM value (2.70 × 10^−5^ M) in Equation (11), the IS and f_mix_^50%^ were determined to be 1.675 and 0.626, respectively (Table 1b). The calculated IS of *H. vulgare* was similar to that of *A. fischeri* (f_mix_^50%^ = 0.616) (Table 1b). The IS of wheat *T. aestivum* was also calculated to assess its applicability to other terrestrial plants. Wang et al. [22] reported the EC50 values of *T. aestivum* (toxic endpoint = root elongation change over 48 h) in hydroponic cultures. The calculated IS (f_mix_^50%^) of *T. aestivum* was 0.015 (Table 1b), implying that the sensitivity to As(V) is considerably higher in *T. aestivum* than in *H. vulgare*.

**Figure 3:**
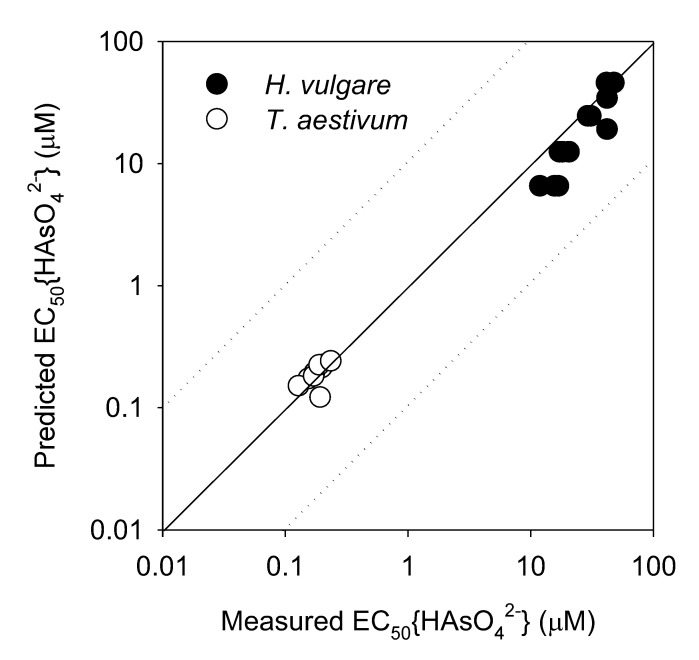
Comparison of measured EC_50_{HAsO_4_^2−^} and predicted EC_50_{HAsO_4_^2−^} by the extrapolated BLM considering Ψ_0_. Open and closed circles indicate the EC_50_{HAsO_4_^2−^} values for *T. aestivum* and *H. vulgare*, respectively. The solid line represents a perfect match between the measured and predicted EC50 values. The dashed lines indicate the difference between the measured and predicted values within an order of magnitude.

The extrapolated BLM to predict As(V) toxicity to *H. vulgare* and *T. aestivum* was validated by comparing the measured EC50 values with the predicted EC50 values (Figure 3). To analyze the effect of Ψ_0_ described in Section 3.1, the slopes of the negative linear relationships (p) in Figure 2a,b were used following Equation (12), when EC50 was predicted.

(12) $${\mathrm{EC}}_{50}{\left\{{\mathrm{HAsO}}_{4}{}^{2-}\right\}}_{\mathrm{site}}={\mathrm{EC}}_{50}{\left\{{\mathrm{HAsO}}_{4}{}^{2-}\right\}}_{\mathrm{ini}}+p\left(\Psi_{0,\mathrm{site}}-\Psi_{0,\mathrm{ini}}\right)$$

where EC_50_{HAsO_4_^2−^}_site_ is the value indicating the effect of Ψ_0_ at a site; EC_50_{HAsO_4_^2−^}_ini_ is the predicted EC50 value from the extrapolated BLM; Ψ_0,site_ is the PM surface electrical potential calculated from the chemical analysis of water at the site; Ψ_0,ini_ is the PM surface electrical potential calculated from the chemical analysis of water in the toxicity test solution when determining the EC50 value.

The model-predicted EC_50_{HAsO_4_^2−^} and experimentally determined EC_50_{HAsO_4_^2−^} exhibited a strong linear relationship within an order of magnitude (Figure 3). This demonstrated that although only IS is considered, site-specific As(V) toxicity to terrestrial plants can be predicted via the chemical analysis of water or porewater with significant accuracy. Thus, the BLM parameters for each plant species are not required, that is, the conditional binding constants between As(V) or phosphate and ABSs of the cell membrane are the same for both species; however, only one EC50 should be used for sensitivity correction. To achieve improved prediction accuracy, the relationship between EC50 values and Ψ_0_ must be derived.

As(V) is assimilated into cells by the existing orthophosphate transporters in both prokaryotes and eukaryotes [28,29], because As(V) is a chemical analog of orthophosphate. This suggests that there are no severe problems in directly using the BLM parameters (e.g., conditional binding constants: K_XH2AsO4_, K_XHAsO4_, K_XH2PO4_, and K_XHPO4_) derived from *A. fischeri* to predict As(V) toxicity to terrestrial plants. The BLM for As(V), originally developed for *A. fischeri*, can be extrapolated to terrestrial plants, including *H. vulgare* and *T. aestivum* across, plant kingdoms.

## 4. Conclusions

In this study, the toxicity caused by As(V) to the terrestrial plants, namely, *H. vulgare* and *T. aestivum*, was predicted using the existing BLM for bioluminescence *of A. fischeri* via interspecies extrapolation. The EC_50_{HAsO_4_^2−^} values decreased from 45.1 ± 4.34 to 15.0 ± 2.60 μM as Ca^2+^ concentration increased from 0.2 to 20 mM, owing to the accumulation of H_2_AsO_4_^−^ and HAsO_4_^2−^ on the PM surface. This was successfully predicted in the extrapolated BLM using a linear relationship between Ψ_0_ and EC_50_{HAsO_4_^2−^}. Consequently, the BLM for As(V) developed for a single bacterium can predict As(V) toxicity to terrestrial plant species among various physicochemical properties by optimizing IS.

## Figures and Tables

**Table 1:** (a) Conditional binding constants of the biotic ligand model (BLM) for inorganic arsenate (As(V)) obtained from *A. fischeri* [14] and (b) calculated inherent sensitivity (f_mix_^50%^) of *A. fischeri* and terrestrial plants.

(a) Parameters of BLM for As(V) obtained from An et al. [14]				
Conditional binding constant ^a^	log K_XH2AsO4_	log K_XHAsO4_	log K_XH2PO4_	log K_XHPO4_
Value (L/mol)	3.067	4.802	3.424	4.588
(b) Inherent sensitivity of *A. fischeri* and terrestrial plants				
Species		Toxic endpoint		f_50_ (dimensionless)
*Aliivibrio fischeri*		5 min bioluminescence inhibition		0.616 ^b^
*Hordeum vulgare*		5 days relative root elongation		0.626 ^c,d^
*Triticum aestivum*		2 days relative root elongation		0.015 ^c,e^

^a^ Conditional binding constants for As(V) (HAsO_4_^2−^ and H_2_AsO_4_^−^) or phosphate (HPO_4_^2−^ and H_2_PO_4_^−^) bound to active binding sites on the cell membrane surface of *A. fischeri*; ^b^ obtained from An et al. [14]; ^c^ Calculated in this study; ^d^ Toxicity data obtained from this study; ^e^ Toxicity data obtained from Wang et al. [22].

## Data Availability

Data is contained within the article.
